# Brain-derived neurotrophic factor polymorphism Val66Met protects against cancer-related fatigue

**DOI:** 10.1038/s41398-020-00990-4

**Published:** 2020-08-26

**Authors:** Li Rebekah Feng, Paul Juneau, Jeniece M. Regan, Josephine Liwang, Sarah Alshawi, Angela Wang, Leorey N. Saligan

**Affiliations:** 1 National Institute of Nursing Research, National Institutes of Health, Bethesda, MD USA; 2 Division of Biostatistics, NIH Library, Office of Research Services, National Institutes of Health, Bethesda, MD USA; 3 Contractor- Zimmerman Associates, Inc., Fairfax, VA USA

**Keywords:** Predictive markers, Clinical genetics, Molecular neuroscience, Depression

## Abstract

Cancer-related fatigue is an extremely common and debilitating psychiatric symptom that affects up to 80% of cancer patients. Despite its negative impact on the patient’s quality of life, there is no well-established biomarker or mechanisms associated with this debilitating condition. The functional brain-derived neurotrophic factor (BDNF) Val66Met single nucleotide polymorphism (SNP) has been associated with a variety of psychiatric illnesses. We hypothesized that Val66Met may influence the risk for developing cancer-related fatigue. BDNF Val66Met was analyzed by polymerase chain reaction in 180 patients with confirmed cancer diagnoses. Fatigue was measured using the Functional Assessment of Cancer Therapy-Fatigue (FACIT-Fatigue) questionnaire. Depression was measured using the Hamilton Depression Scale (HAM-D). Data were transformed when necessary and regression models were constructed to access the association between genotype and symptom severity. Participants carrying the Met allele reported significantly less fatigue compared to the Val/Val genotype group. The presence of the Met allele did not influence depression levels. The results suggest that the BDNF Val66Met polymorphism confers protective advantage against cancer-related fatigue; whereas having the Val/Val genotype may be a genetic risk factor. Findings from this study not only provide clues to the neural basis of cancer-related fatigue, but also allow for symptom severity prediction and patient education with the goal to improve symptom management.

## Introduction

Oncology patients often experience psychiatric symptoms such as cancer-related fatigue, which differs from the normal sense of “tiredness” as it cannot be resolved by rest and severely impacts their daily functioning^1^. Of all cancer-related symptoms, fatigue is often the most distressing and prevalent symptom reported by up to 80% of oncology patients^1,2^. Currently, the diagnosis of cancer-related fatigue relies entirely on self-reports with no well-established biomarkers^3^. In addition, little is known about its underlying mechanisms and no FDA-approved therapeutic interventions are available^1^. As a result, the National Cancer Institute has identified cancer-related fatigue as a first-tier, high-priority research area^4,5^.

The unrelenting sense of tiredness can be both persistent and severe, directly affecting the patient’s ability to perform daily tasks, resulting in helplessness and despair^6^. While there is a scarcity of empirical evidence for effective management strategies, recognition of the symptom by healthcare providers and patient education may alleviate the emotional burden associated with cancer-related fatigue^6^. While cancer-related fatigue is often reported by patients as a multidimensional symptom, the overall sense of tiredness notably exhibits a cognitive component^7,8^. Interestingly, not all patients with cancer or those receiving cancer treatments experience profound fatigue, and it is conceivable that genetic vulnerabilities may alter the threshold of symptom presentation in response to inflammation induced by cancer or its treatments^9^.

Brain-derived neurotrophic factor (BDNF) is a key neurotrophin expressed ubiquitously throughout the brain and plays a central role in almost all facets of brain function such as neuroplasticity, developmental processes, synaptogenesis, and maintenance of functional neuronal networks^10^. BDNF is produced in the neuronal cell body and transported in secretory vesicles into somatodendritic compartments where they are released upon electric stimulation^11^. BDNF is first synthesized as the precursor proBDNF (~32 kDa) and cleaved into mature BDNF (~13 kDa)^12^. Pro-BDNF and mature BDNF (mBDNF) are thought to have opposing effects mediated by specific neurotrophin/receptor interactions: (1) mBDNF dimerizes and binds to tropomyosin-related kinase B (TrkB) receptor and promotes long-term potentiation, neurite outgrowth, and cell survival; (2) proBDNF, on the other hand, promotes long-term depression, growth cone retraction, and neuronal cell death by interacting with pan-neurotrophin receptor 75 (p75^NTR^) and a co-receptor, sortilin^13^.

A particular single nucleotide polymorphism (SNP) that has garnered increasing interest is the BDNF Val66Met (rs6265), a single nucleotide substitution (G196A) causing an amino acid change at codon 66 from the canonical valine (Val) to methionine (Met) in the pro-region of the BDNF^11^. The Met66 allele occurs in 50% of Asians, 30% of Caucasians, and 5% of African Americans^14^. Functionally, Val66Met has been shown to reduce activity-dependent release, but not constitutive release, of BDNF^11^. Even though the Val→Met substitution at codon 66 does not alter the structural or functional properties of mBDNF, Val66Met markedly reduces the association of BDNF with secretory vesicle markers, suggesting that the decrease in activity-dependent release is a result of altered secretion instead of production^11^. The consequences of having the Met allele (Val/Met, Met/Met) have been associated with a plethora of detrimental effects including decreased hippocampal volume, memory deficits, increased anxiety behavior, and increased risk for depression both in animal models and human studies^15–17^. However, the high natural occurrence of Val66Met suggests that the Met allele may confer selective advantage. In fact, the Met allele appears to be protective in older adults: compared to Val/Val individuals, Met allele carriers have demonstrated higher cortical thickness, better episodic memory performance, slower cognitive decline, and decreased risk for Alzheimer’s disease in old age, but not early in adult life^18,19^. In addition, the effect of Val66Met differs based on gender and alters the risk for depression in a gender-specific manner^20^. Val66Met also influences disease outcome in a time-dependent fashion: for example, Met allele carriers exhibited greater acute deficits, but enhanced recovery, after ischemic stroke^21^.

Previous studies on the role of BDNF in cancer-related fatigue relied on measuring BDNF concentrations in plasma or serum as a proxy^22^. However, circulating BDNF is largely attributable to platelet precursor cells, megakaryocytes, which synthesize BDNF under the platelet BDNF promoter^23^. It is not clear whether circulating BDNF measured in blood reflects brain BDNF levels^24^. Despite the importance of BDNF on brain functions and increasing interest in Val66Met on psychiatric/cognitive symptoms, studies on the effects of Val66Met on cancer-related fatigue are non-existent. One study found a higher rate of suicide in cancer patients carrying the Met allele^25^. Interestingly, several recent studies provided new evidence to suggest a protective role of Val66Met against chemotherapy-induced cognitive impairment and depression^26–28^. In addition, Val66Met is a particularly interesting candidate SNP to examine for cancer-related fatigue due to the well-established symptomatic and possible mechanistic overlap between fatigue and depression^29^, and for the profound influence Val66Met has on the risk of depression in otherwise healthy individuals^17^.

The goal of this study was to determine the role of the BDNF Val66Met genotype on cancer-related fatigue in subjects with confirmed cancer diagnoses. As Val66Met has been shown to increase the risk for depression in healthy individuals^29^, we also examined whether this SNP influences depression in cancer patients. Here we report that Val66Met confers protective advantage against developing cancer-related fatigue. However, in contrast with healthy individuals, the risk for the development of depression is not altered by Val66Met in subjects with cancer.

## Materials and methods

### Participants

Data were collected from two studies: (1) patients with non-metastatic prostate adenocarcinoma scheduled to receive radiotherapy (NCT00852111); (2) patients with any type of cancer scheduled to receive any type of treatment (NCT01231932). Signed written informed consents were obtained prior to study participation.

NCT00852111 was approved in 2008 by the Institutional Review Board (IRB) of the National Institutes of Health (NIH). All subjects were 18 years of age or older, with confirmed cancer diagnoses, and scheduled to start cancer treatment at the NIH. Prospective participants were excluded if they had an unstable or end-stage disease of any body system, uncorrected hypothyroidism, untreated anemia, any medical history of tuberculosis, any infectious disease such as HIV or hepatitis, a chronic inflammatory disease, or a psychiatric disorder diagnosis within the past 5 years, or a second malignancy. Those receiving chemotherapy or taking medications known to affect cytokine production, such as tranquilizers, steroids, and nonsteroidal anti-inflammatory agents, were also excluded from this study.

### Instruments

Fatigue was measured using the Functional Assessment of Chronic Illness Therapy-fatigue (FACIT-Fatigue) scale. The total fatigue scores range from 0–52; lower scores indicate higher fatigue intensity. Subjects with a FACIT-Fatigue score <43 were considered fatigued^30,31^. A 3-point difference in FACIT-Fatigue scores is considered to be a minimally clinically important difference^32–34^. Depression was measured using the Hamilton Depression Rating Scale (HAM-D). High scores indicate more severe depression: a score of 0–7 indicates no depression, a score of 8–16 indicates mild depression, and a score of ≥17 indicates moderate-to-severe depression^35^. See supplementary methods for more details.

### BDNF rs6265 profiling

Whole blood samples were collected in ethylene-diamine-tetra-acetic acid tubes (BD Biosciences, Franklin Lakes, NJ, USA) and extracted using PureLink^™^ Pro 96 Genomic DNA Purification Kit (Invitrogen, Carlsbad, CA, USA). The region containing the BDNF Val66Met SNP (GenBank dbSNP: rs6265) was profiled using polymerase chain reaction (PCR) using the following primers: forward 5′–AGAAGAGGAGGCTCCAAAGG–3′ and reverse 5′–ACAAGGTGGCTTGGCCTAC–3′. After enzymatic purification, sequencing was performed by using BigDye^™^ Terminator Cycle Sequencing Kit (ThermoFisher Scientific, Waltham, MA, USA). Data analysis was performed using the DNASTAR Lasergene12^®^ software (DNASTAR, Inc, Madison, WI, USA). The threshold for SNP detection was set to 10%. Mutations from the reference sequence were called when possible sequence quality and coverage allowed.

### Statistics

Demographic and clinical characteristics were summarized by standard descriptive statistics. Values are expressed as Mean ± Standard Deviation (SD). For comparisons of clinical characteristics between groups, Student’s two-sample *t* test, Dunnett’s test, and the Wilcoxon Rank Sum test were used when appropriate and a *chi*-squared test was employed to examine differences in proportions. Using power analysis with an alpha of 0.05 and power of 80%, the projected sample size needed is ~41 subjects per group and a total of 82 subjects.

The first measurement of interest was the FACIT-Fatigue score and the chief predictor was genotype. The FACIT-Fatigue score was bounded at the upper limit by a maximum score of 52, inducing an asymmetrical distribution pattern that is left-skewed. To accommodate this feature, each score was divided by the maximum score, and then transformed with a logistic transformation^36^. The resultant transformed data had reduced skewness, affording the use of traditional statistical methods. The transformed FACIT-Fatigue score was first modeled as a function of genotype, and then modeled as a function of genotype, patient age, and a corresponding interaction (joint) effect of both within a generalized linear model assuming a normal distribution for the transformed outcome and an identity link.

As a larger proportion of total HAM-D scores equaled to zero (no depression) than would be predicted by standard probability models, the fit of zero-inflated models was explored. The total score could be viewed as a count; thus, zero-inflated Poisson^37^ and zero-inflated negative binomial^38^ models were employed to compare the scores between the two genotypes. A bootstrapped 95% confidence interval was estimated for the difference in the scores between the two genotypes. Model assessment was performed by comparing the outcome value frequencies against their corresponding observed frequencies. If the observed fit was similar between the zero-inflated Poisson and the negative binomial models, the probability model with the fewest parameters was selected. As was the case with the FACIT-Fatigue score, a second model was fit with terms for genotype, age, and their joint (interaction) effect.

Mean HAM-D and FACIT-Fatigue scores for those patients with T stage T1 were compared with all other stages by Dunnett’s test. The application of Dunnett’s test afforded protection against an inflation of the experiment-wise error rate for these two families of comparisons. All statistical tests were two-sided and conducted at the 0.05 level of statistical significance. Statistical analyses were performed using JMP 15.0.0 (SAS Institute, Cary, NC, USA) and SAS Version 9.4 (TS1M6, SAS Institute).

## Results

### Clinical characteristics

A total of 180 cancer patients were recruited for the study. To eliminate the potential confound introduced by the interaction between Val66Met and gender, we only included male subjects in the current study. The sample cohort were older men with a mean age of 65.3 ± 8.1 years (Table 1). Participants were predominantly Caucasian (73.3%). Prostate adenocarcinoma accounted for 95.5% of the cancer types (Table 1). Cancer T stages ranged from T1 to T4 (Table 1). Subjects with T0 or Tx at the time of the study were only included in the study if additional confirmatory cancer diagnostic evidence was obtained.

**Table 1:** Clinical characteristics (values are mean ± standard deviation).

		*N* (%)
Age (years)	65.3 ± 8.1	
Race	Caucasian	132 (73.3)
	African American	34 (18.9)
	Asian	9 (5.0)
	Hispanic	3 (1.7)
	Other	1 (0.6)
	Unknown	1 (0.6)
Height (cm)	175.7 ± 7.7	
Weight (kg)	88.5 ± 14.7	
Body mass index (kg/m^2^)	28.7 ± 4.4	
Hemoglobin (g/DL)	14.1 ± 1.2	
Education	Less than high school	1 (0.6)
	High school	20 (11.1)
	Associate degree/some college	10 (5.6)
	College/technical/vocational school	86 (47.8)
	Graduate school/postgraduate	61 (33.9)
	Unknown	2 (1.1)
T stage	T0	7 (3.9)
	T1c	63 (35.0)
	T2a–c	62 (34.4)
	T3a–b	37 (20.6)
	T4	5 (2.8)
	Tx	6 (3.3)
Cancer type	Prostate Adenocarcinoma, non-metastatic	163 (90.6)
	Prostate Adenocarcinoma, metastatic	7 (3.9)
	Prostatic Adenocarcinoma (ductal), non-metastatic	2 (1.1)
	Choroidal Tumor, non-metastatic	1 (0.6)
	Epithelioid Uveal Melanoma Liver, metastatic	1 (0.6)
	Lung Adenocarcinoma, non-metastatic	1 (0.6)
	Colon Adenocarcinoma, metastatic	1 (0.6)
	Pancreatic Adenocarcinoma, metastatic	1 (0.6)
	Neuroendocrine Carcinoma, non-metastatic	1 (0.6)
	Ocular Melanoma, non-metastatic	1 (0.6)
	Pancreatic Cancer, non-metastatic	1 (0.6)
	Urothelial Carcinoma, non-metastatic	1 (0.6)
	Unknown	3 (1.7)

The BDNF Val66Met SNP genotype frequencies of the entire cohort were in Hardy–Weinberg Equilibrium (*χ*^2^ = 0.45, *p* = 0.504). The common homozygous Val/Val accounted for 66.11% of the total sample. Val/Met heterozygous and Met/Met homozygous represented 29.44% and 4.44% of the total sample, respectively (Table 2).

**Table 2:** Genotype and allele frequencies of the BDNF Val66Met polymorphism (rs6265).

Genotype	Absolute frequency	Relative frequency %
GG (Val/Val)	119	66.11%
AG (Val/Met)	53	29.44%
AA (Met/Met)	8	4.44%
G (Val) allele	291	80.83%
A (Met) allele	69	19.17%

### Val66Met alters risk for fatigue but not depression in cancer patients

**Fig. 1:**
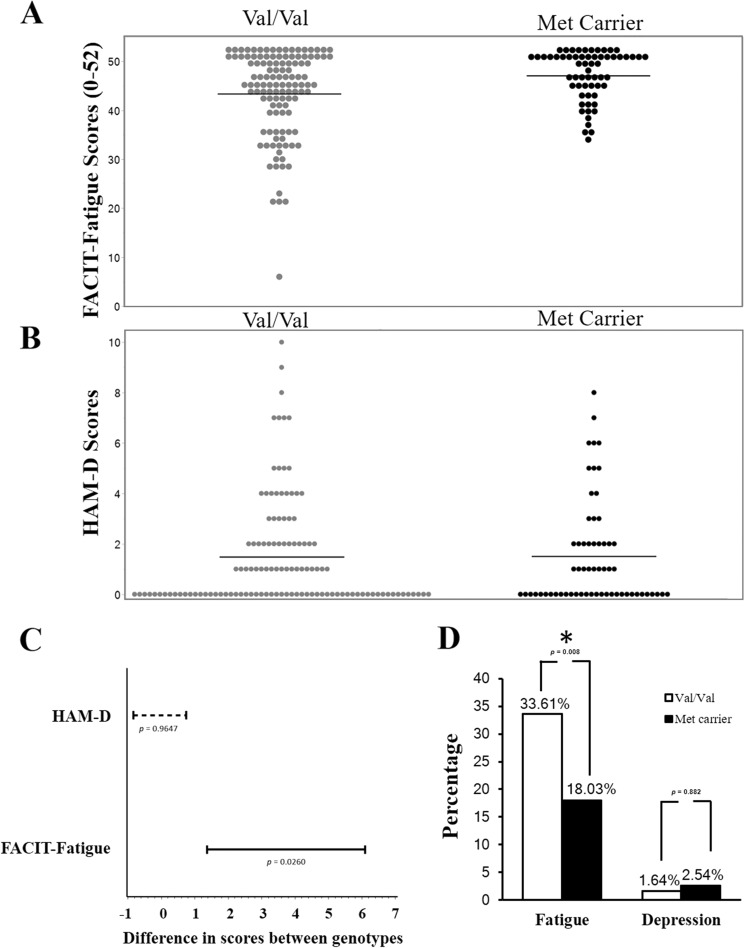
BDNF Val66Met affected fatigue, but not depression, in cancer patients. **a** Scatter plot of FACIT-Fatigue scores grouped by genotype. FACIT-Fatigue scores of subjects that carried the Met allele (47.089 ± 4.944) compared to subjects with Val/Val (43.357 ± 8.654) were significantly higher (*F*_1178_ = 9.70, *p* = 0.002). **b** Scatter plot of HAM-D scores grouped by genotype. There was no difference (*F*_1177_ = 0.00, *p* = 0.961) between subjects that carried the Met allele (1.508 ± 2.087) compared to subjects with Val/Val (1.492 ± 2.171). **c** Confidence Interval (CI) plot demonstrating that the difference between genotypes (Met carrier vs. Val/Val) was only significant in FACIT-Fatigue scores (*p* = 0.026) but not in HAM-D scores (*p* = 0.965). **d** The percentage of fatigued subjects (FACIT-Fatigue score <43), in the Val/Val group was significantly higher (33.61%) than the Met carrier group (18.03%) (*p* = 0.008, Wilcoxon Rank-Sums test). The rate of depression (HAM-D score ≥8) was not significant between the two genotypes (1.64 vs. 2.54%; *p* = 0.882).

Met carriers (Met/Met and Val/Met) reported significantly higher FACIT-Fatigue scores indicating lower fatigue levels (47.1 ± 4.9) compared to Val/Val homozygous (43.4 ± 8.7) (Fig. 1a; *F*_1178_ = 9.70, *p* = 0.002). On the other hand, HAM-D scores were not significantly different between genotypes (Fig. 1b; Met carrier: 1.492 ± 2.171 vs. Val/Val: 1.508 ± 2.087; *F*_1177_ = 0.00, *p* = 0.961).

A regression model was constructed to analyze FACIT-Fatigue scores as a function of genotype, and the adequacy of the fit of the model was shown in supplementary Fig. 1. The distribution of the model’s studentized deviance residuals were symmetrical and could be approximated by a standard Gaussian curve; thus allowing for the construction of a generalized linear model assuming a normal distribution for the transformed outcome (supplementary Fig. 1A). The symmetry and homoscedasticity of the model were further demonstrated by the agreement between the percentiles of a standard Gaussian distribution and the cumulative distribution of the Studentized deviance residuals (supplementary Fig. 1B). Carrying the Met allele significantly predicted lowered risk for fatigue at a 95% confidence interval (Table 3; 95% CI [0.0769, 1.2117], *p* = 0.026).

**Table 3:** Regression model of FACIT-Fatigue scores as a function of genotype.

Analysis of maximum likelihood parameter estimates - fatigue							
Parameter	DF	Estimate	Standard error	Wald 95% confidence limits		Wald χ2	Pr > χ2
Intercept	1	2.4214	0.1685	2.0911	2.7517	206.42	<0.0001
rs6265 Met carrier	1	0.6443	0.2895	0.0769	1.2117	4.95	0.0260*
rs6265 Val/Val	0	0.0000	0.0000	0.0000	0.0000	–	–

*Indicates statistical significance, *p* < 0.05.

As most of the study participants were not depressed, a larger proportion of “0” HAM-D scores were reported than would be predicted by standard probability models. Zero-inflated Poisson and zero-inflated negative binomial models were thus constructed to compare the difference between genotypes in HAM-D scores (Supplementary fig. 2). The model predicted “0” values almost perfectly and positive values by <8% points of deviation (Supplementary Fig. 2A). The deviations of zero-inflated Poisson predicted values from observed HAM-D values by only 0.5–8% points indicating a sound fit of the model (Supplementary Fig. 2B). Carrying the Met allele did not predict the risk for depression (Table 4; 95% CI [−0.2726, 0.2852], *p* = 0.965).

**Table 4:** Regression model of HAM-D scores as a function of genotype.

Analysis of maximum likelihood parameter estimates - depression							
Parameter	DF	Estimate	Standard error	Wald 95% confidence limits		Wald χ2	Pr > χ2
Intercept	1	1.0240	0.1154	0.7979	1.2502	78.79	<0.0001
rs6265 Met carrier	1	0.0063	0.1423	−0.2726	0.2852	0.00	0.9647
rs6265 Val/Val	0	0.0000	0.0000	0.0000	0.0000	–	–

The difference between Met carriers and Val/Val subjects in FACIT-Fatigue scores was statistically significant (Fig. 1c; 95% CI [1.37, 6.10], *p* = 0.026). In contrast, the corresponding 95% CI for the difference in mean HAM-D scores between the two cohorts [−0.84, 0.74] was not statistically significant (*p* = 0.965). We observed a significantly higher percentage of fatigued subjects (FACIT-Fatigue score <43) in the Val/Val group (33.6%), whereas only 18.0% of Met carriers were fatigued (*p* = 0.008). The rate of depression was low in both groups (Val/Val: 1.64% vs. Met carrier: 2.54%) and the difference between genotypes was not significant (Fig. 1d; *p* = 0.882).

Regardless of the cancer type, participants carrying the Met allele generally fell within the top quartile of the FACIT-Fatigue distribution, whereas participants with two copies of Val fell within the bottom quartile (Supplementary Fig. 3). Dunnett’s test was conducted to compare FACIT-Fatigue and HAM-D scores among T stages; no significant difference was detected at a 0.05 nominal level of statistical significance (Supplementary Fig. 4).

Participants carrying the Met allele exhibited lower body mass index (BMI) compared to the Val/Val group (Supplementary Fig. 5A; *F*_1153_ = 6.02, *p* = 0.016). BMI weakly correlated with actigraphy daily activity count (adjusted *R*^2^ = 0.05, *p* = 0.006). However, we did not observe any difference in daily activity count between genotypes (*F*_1156_ = 0.01, *p* = 0.910). Furthermore, daily activity count in the current study did not correlate with FACIT-Fatigue scores (Supplementary Fig. 5A; adjusted *R*^2^ = 0.0002, *p* = 0.311).

## Discussion

Multiple lines of evidence suggest that cancer-related fatigue may be a result of unresolved inflammation stemming from a combination of genetic risk factors and inflammatory triggers including cancer^9,39^. Even though cancer-related fatigue represents a general sense of tiredness, emerging evidence points toward a cognitive component that involves a potential alteration in brain connectivity^8,40^. The Val66Met polymorphism within the pro-domain of BDNF has been associated with a variety of psychiatric disorders including depression, a symptom that is often co-morbid with cancer-related fatigue^17,29^. To our knowledge, this is the first study to demonstrate a novel genetic association between BDNF Val66Met polymorphism and lowered risk for developing cancer-related fatigue. Interestingly, although Val66Met has been shown to greatly increase the risk for depression in healthy individuals^29^, carrying the Met allele did not affect depression levels in patients with cancer. These findings are clinically significant as Val66Met may serve as a novel genetic marker that specifically predicts the risk for cancer-related fatigue, but not the often-comorbid depressive symptoms. In addition, our study offers a first glimpse into the neural basis of cancer-related fatigue and provides clues to developing future therapeutic interventions.

A growing body of literature suggests a causal role of peripheral inflammation in cancer-related fatigue pathogenesis as a result of either cancer or cancer treatment^9,39^. Although the exact mechanisms whereby peripheral inflammation results in fatigue symptoms is unknown; immune-chemosensory signaling via the afferent vagus nerve is thought to transduce peripheral inflammation signals to the central nervous system^41^. In fact, vagus nerve transection in an animal model of inflammation attenuated sickness behavior, which includes fatigue^42^. Interestingly, healthy adults with the Met/Met genotype exhibit reduced vagal activity as well as reduced cortisol response to acute stress^43,44^. It is possible that the Met allele confers advantage by dampening the hyperactivation of the vagus nerve immune sensory signaling pathway and decreases the risk for the consequent cancer-related fatigue.

Another possible explanation for the protective advantage of the Met allele may be related to the upregulation of proBDNF under pathological conditions^45^ combined with downregulated TrkB transcription and unchanged p75^NTR^ in old age^46,47^. This suggests a shift in balance between the two forms of BDNF toward a more proapoptotic effect mediated by proBDNF, which may be particularly detrimental to patients with advanced age like those in the current patient cohort. Since the Val66Met genotype is associated with reduced activity-dependent secretion of both pro- and mature BDNF, it is conceivable that carrying the Met allele affords protection against cancer-related fatigue by reducing the proapoptotic effects of proBDNF^48^. It is worth noting that the protective advantage of Val66Met is likely to be genetic rather than a result of altered physical activity, as we did not observe any difference in daily activity between genotypes (Supplementary Fig. 5).

This study has several limitations. First, our study included subjects with multiple types of cancer with various levels of severity. Our decision not to exclude cancer types with small sample sizes was because patients will likely seek care because of the negative impact of fatigue on daily living regardless of the type of cancer. Interestingly, neither the cancer type nor the disease severity altered our findings. It is conceivable that Val66Met could be used as a general predictor for lowered risk of cancer-related fatigue, regardless of the cancer type or severity. We are actively recruiting patients with various types of cancer to test this hypothesis. Secondly, previous studies have shown that cancer and cancer treatment may lead to fatigue via different mechanisms^39,49,50^. We only included participants that had confirmed cancer diagnoses before the start of treatment in order to focus on fatigue related to cancer itself instead of cancer treatment. Future work will explore the effect of Val66Met on longitudinal changes in fatigue symptoms before and after cancer treatment. Thirdly, all participants included in this study were male to avoid the confound of gender-specific effects of BDNF^51^. Current efforts are being made to recruit female participants to further examine whether this polymorphism affects cancer-related fatigue and depression differently in different genders. In addition, future studies with a larger sample size will explore whether sex hormones influence the effect of Val66Met on fatigue. It is worth noting that BMI, which has been shown to increase the risk for psychiatric conditions such as depression and anxiety^52^, did not significantly correlate with fatigue or depression levels in our study. It is possible that there exists a ceiling effect where neuroinflammation related to cancer is already at a level whereby any additional contribution by BMI was not detected. Future studies that include non-cancer patients will help elucidate this point. Finally, the rationale for grouping Met heterozygous and homozygous individuals as Met carriers was based on in vitro evidence demonstrating the similarity in secreted BDNF between these genotypes^53^. While the sample size in the current study did not allow for comparisons among Val/Val, Val/Met, and Met/Met, future studies will investigate whether differences in protection against fatigue exist between Met heterozygous and homozygous individuals.

Clinical application of Val66Met SNP as a prognostic/predictive marker for assessing the risk of developing cancer-related fatigue has several advantages: (1) SNP detection can be easily performed requiring only a small sample of blood; (2) genotypic marker detection is affordable, reliable, and yields reproducible results; (3) using the functional Val66Met polymorphism provides a more accurate assessment for brain BDNF and bypasses the aforementioned disadvantage of measuring serum/plasma BDNF concentrations, which is largely attributable to platelet-derived BDNF. Furthermore, the knowledge of a patient’s Val66Met genotype could thus result in a more precise treatment recommendation for that patient, given his or her disease circumstances. For example, lower levels of daytime activity has been associated with worse cancer-related symptoms including depression and fatigue^54^. Exercise is often recommended for patients suffering from cancer-related fatigue, and exercise has been shown to increase both proBDNF and mBDNF^55–57^. It is conceivable that exercise should be prescribed to fatigued patients differently based on the Val66Met genotype, particularly if abnormalities in proBDNF production or signaling may play a role in the pathogenic process of the symptom.

## Supplementary information

Supplemental material

## Data Availability

The datasets used and/or analyzed during the current study are available from the corresponding author on reasonable request.
