# Supplementary material for: Brain-derived neurotrophic factor polymorphism Val66Met protects against cancer-related fatigue
Source: Transl Psychiatry. 2020 Aug 26;10:302. doi: 10.1038/s41398-020-00990-4 (PMC7450091; doi:10.1038/s41398-020-00990-4)
Supplement: Supplementary file 1 — Supplemental material [file 41398_2020_990_MOESM1_ESM.docx]

**Supplemental methods:**

**Instruments**

Fatigue was measured using the 13-item Functional Assessment of Chronic Illness Therapy-fatigue (FACIT-Fatigue) scale, a validated and reliable instrument specifically developed to measure cancer-related fatigue.^1^ FACIT-Fatigue has demonstrated good internal consistency reliability (Cronbach’s α= 0.81) in the current study cohort. FACIT-Fatigue items are scored on a 0–4 scale (0 = “not at all”, 4 = “very much”). The total fatigue scores range from 0–52; lower scores indicate higher fatigue intensity. A FACIT-Fatigue score of 43 best divides fatigue scores of cancer patients and the general population^2^, and subjects with a FACIT-Fatigue score <43 were considered fatigued.^3,4^ A 3-point difference in FACIT-Fatigue scores is considered to be a minimally clinically important difference (MCID), defined as “the smallest difference in score in the domain of interest which patients perceive as beneficial and which would mandate, in the absence of troublesome side effects and excessive cost, a change in patient’s management”.^5-7^

Depression was measured using the 17-item Hamilton Depression Rating Scale (HAM-D). High scores indicate more severe depression: a score of 0–7 indicates no depression, a score of 8–16 indicates mild depression, and a score of ≥17 indicates moderate-to-severe depression.^8^ HAM-D has demonstrated good internal consistency (standardized Cronbach’s α = 0.67–0.80) and test-retest reliability (Pearson correlation coefficient = 0.88, *p* < 0.001).^9^

**Actigraphy**

A hip-mounted piezoelectric accelerometer device was used to quantify physical activity at 1-second intervals (Actical^®^; Philips Healthcare, Bend, OR, USA). The accelerometer activity measurement is proportional to the degree and intensity of the movement. The area under the integrated activity curve is stored as “activity counts,” which are then summed for each 15-second long epoch and valued at zero in the absence of activity.^10^ Participants were instructed to wear the device for 4 days, of which two 24-hour periods starting from 2 consecutive activity counts on a weekday were gathered. Total daily activity count was the sum of all activity counts during a 24-hour period to provide an activity count per day.^11^

**Supplemental Figure 1:**

Model adequacy of the fit of FACIT-Fatigue total score by genotype. **(A)** The distribution of model’s studentized deviance residuals. Note that the Studentized deviance residuals are symmetrical about their mean and could be approximated by a standard Gaussian or normal curve (compare the nonparametric kernel estimate with fitted normal distribution). **(B)** A P×P Plot of the model’s Studentized Deviance Residuals. Further adequacy of the model is established by the agreement between the percentiles of a cumulative standard normal (Gaussian) distribution and the cumulative distribution of the Studentized deviance residuals.


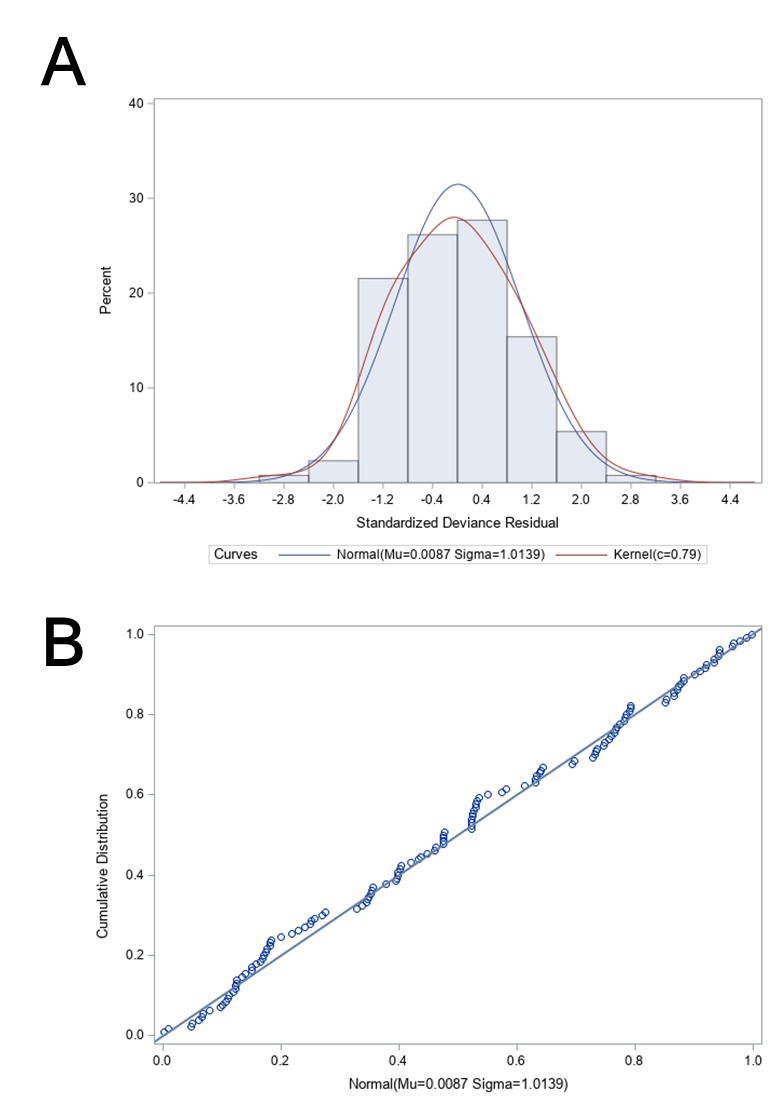


**Supplemental Figure 2:**

Model adequacy of the fit of HAM-D score by genotype. **(A)** A comparison of the relative frequency of the observed scores by those predicted by the Zero-Inflated Poisson (ZIP) model. The fitted values, predicted by the ZIP model, are compared with the relative frequencies of the HAM-D scores observed for the participants included in the study. Note the model predicts the frequency of patients with scores equal to zero nearly perfectly. The deviations between the predicted and observed for positive values of the score are close, and deviate, at most, by approximately 8 percentage points. (**B)** A comparison of the relative frequency of the observed scores by those predicted by the ZIP model by a Plot of the Deviations. The plot shows the observed HAM-D scores amongst the study participants minus those predicted by the ZIP model. Note that the deviation between the two is as small as approximately 0.5 percentage point and, at most, as large as about 8 percentage points.


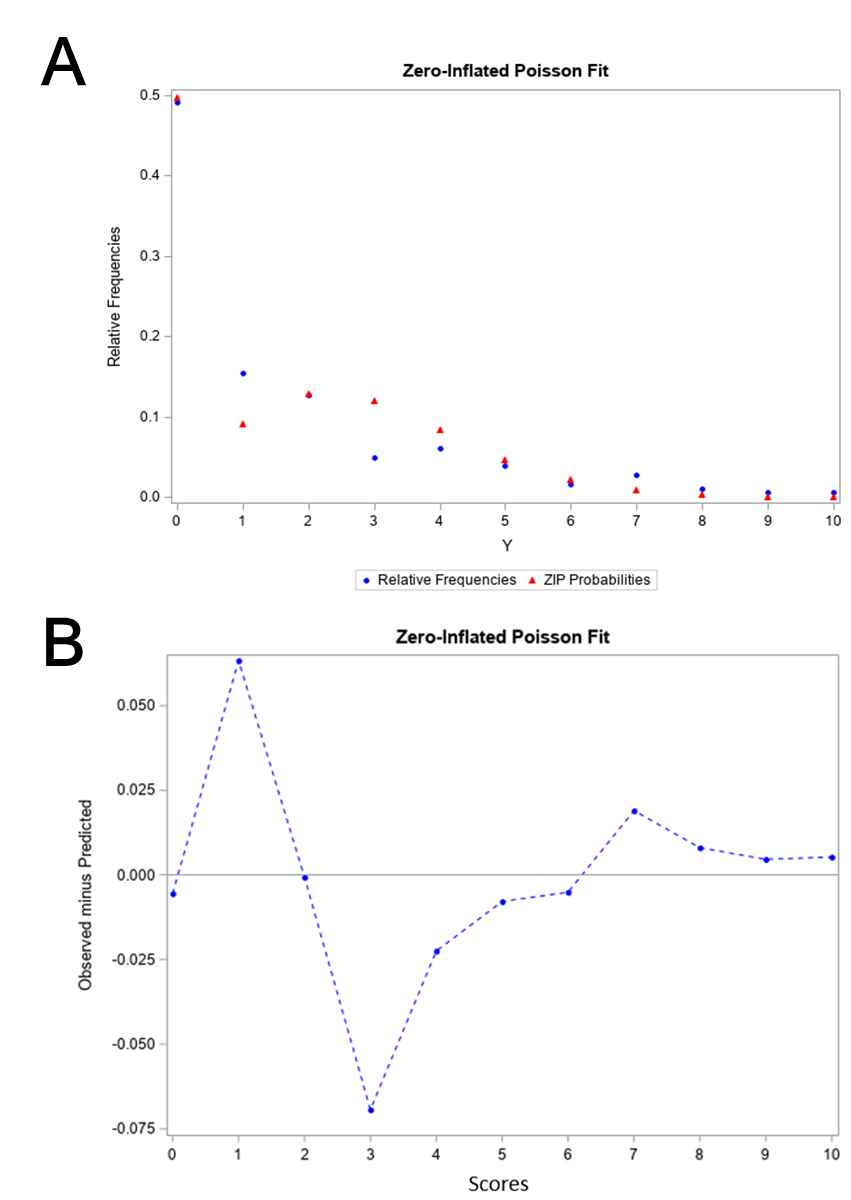


**Supplemental Figure 3:**

**
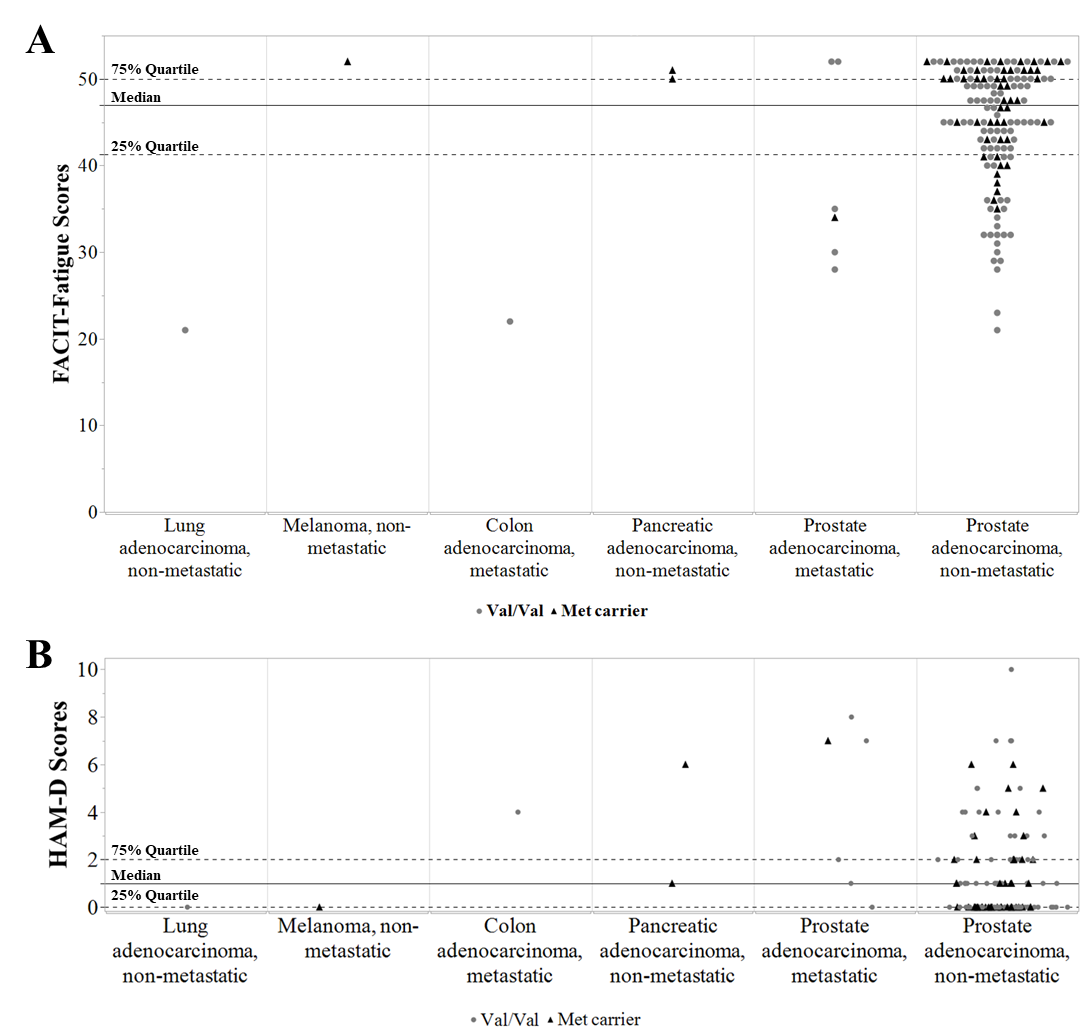
**Cancer types did not alter the pattern of effects of Val66Met on fatigue or depression. **(A)** In non-prostate adenocarcinoma cancer types, the pattern of FACIT-Fatigue scores follow the same pattern as the group–Met carriers were less fatigued and reported higher FACIT-Fatigue scores. **(B)** HAM-D scores among different cancer types and genotypes appear to be randomly distributed. Gray circles indicate Val/Val, black triangles indicate Met carriers.

**Supplemental Figure 4:**

Cancer severity did not appear to alter the pattern of effects of Val66Met on fatigue or depression. Box and whisker plots of the FACT-F **(A)** and HAM-D **(B)** scores, respectively, stratified by genotype (Met carrier vs. Val/Val). Mean patient scores for tumor stages T0, T2, T3, T4, and Tx were compared with the mean score of patients in the T1 stage, without considering a patient’s genotype. An application of Dunnett’s test for these comparisons, conducted a 0.05 nominal level of statistical significance, did not provide evidence of a statistically significant difference in the means for either score.

**
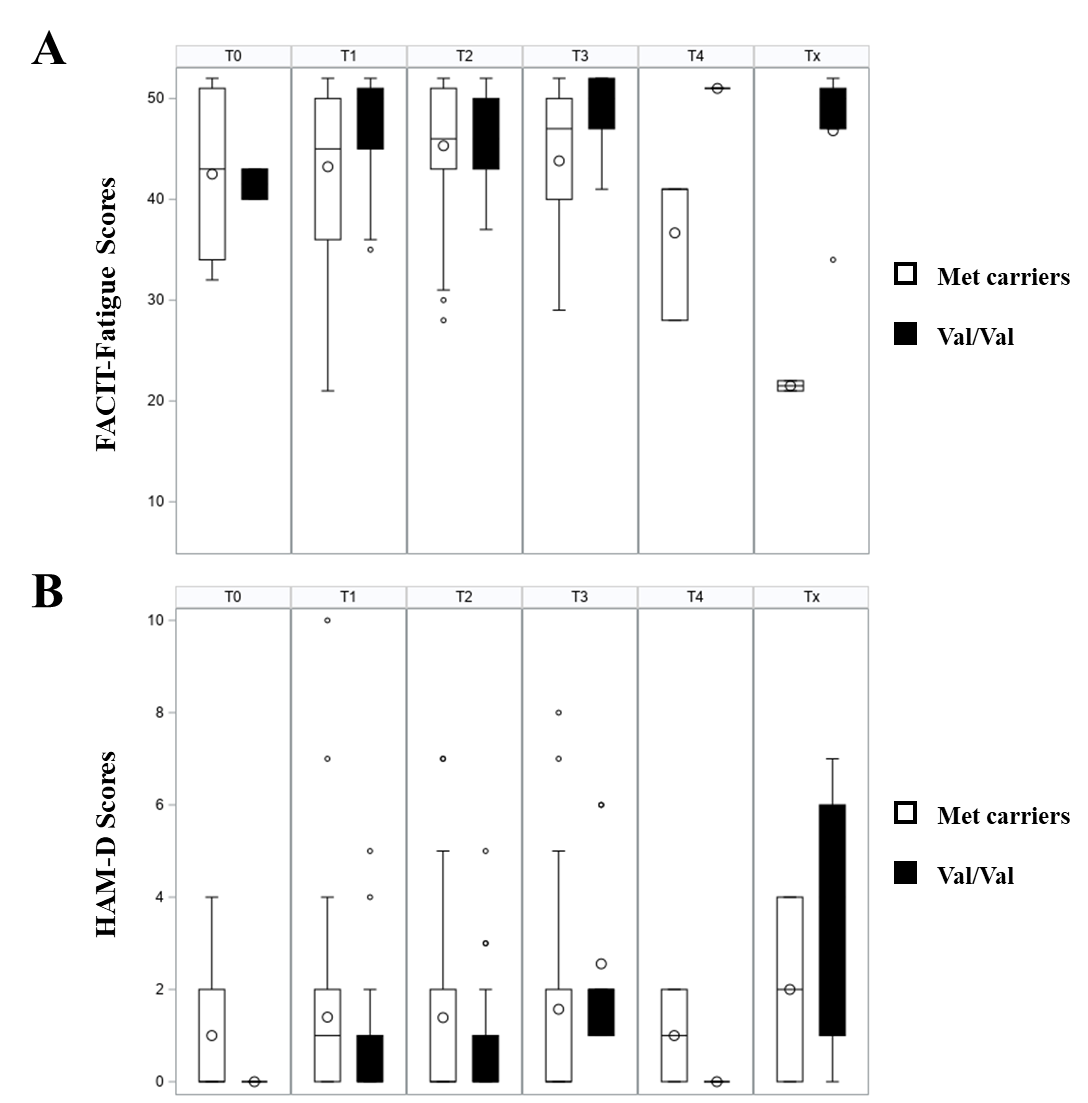
**

**Supplemental Figure 5:**

Val66Met was associated with the body mass index (BMI), but not daily activity count. **(A)** Met carriers had a lower BMI on average compared to Val/Val subjects (*F*_1,153_ = 6.02, *p* = 0.016). FACIT-Fatigue scores correlated with BMI in a weak but statistically significant manner (adjusted *R*^2^ = 0.09, *p* = 0.001). **(B)** No difference was detected in the actigraphy total daily activity count between the genotypes (*F*_1,156_ = 0.01, *p* = 0.910). Even though total daily activity and BMI correlated significantly (adjusted *R*^2^ = 0.05, *p* = 0.006), FACIT-Fatigue did not correlate significantly with average daily activity count (adjusted *R*^2^ = 0.0002, *p* = 0.311).


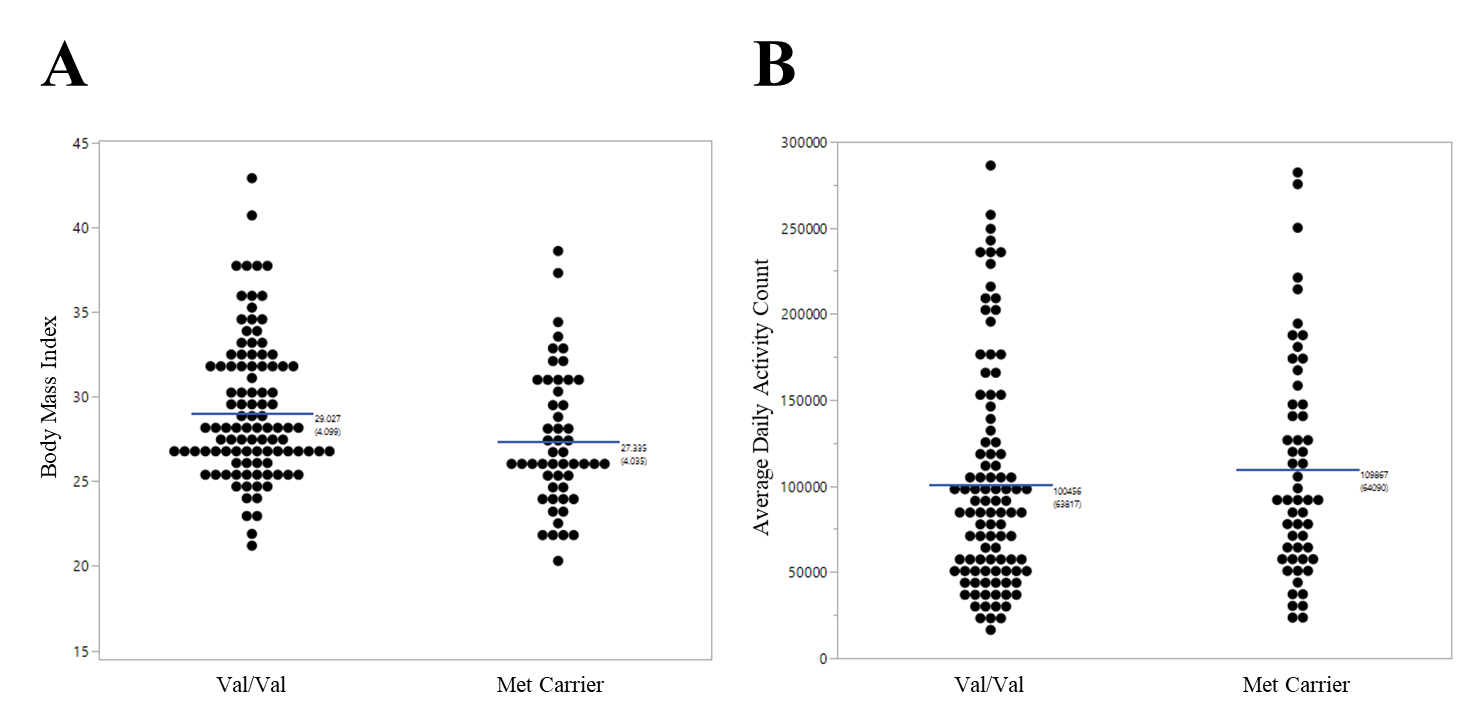


1. Yellen SB, Cella DF, Webster K, Blendowski C, Kaplan E. Measuring fatigue and other anemia-related symptoms with the Functional Assessment of Cancer Therapy (FACT) measurement system. *Journal of Pain and Symptom Management* 1997; **13**(2): 63-74.

2. Cella D, Eton DT, Lai J-S, Peterman AH, Merkel DE. Combining Anchor and Distribution-Based Methods to Derive Minimal Clinically Important Differences on the Functional Assessment of Cancer Therapy (FACT) Anemia and Fatigue Scales. *Journal of Pain and Symptom Management* 2002; **24**(6): 547-61.

3. Spichiger E, Müller-Fröhlich C, Denhaerynck K, Stoll H, Hantikainen V, Dodd M. Prevalence and contributors to fatigue in individuals hospitalized with advanced cancer: A prospective, observational study. *International Journal of Nursing Studies* 2012; **49**(9): 1146-54.

4. Cella D, Lai J-s, Chang C-H, Peterman A, Slavin M. Fatigue in cancer patients compared with fatigue in the general United States population. *Cancer* 2002; **94**(2): 528-38.

5. Yost KJ, Eton DT, Garcia SF, Cella D. Minimally important differences were estimated for six Patient-Reported Outcomes Measurement Information System-Cancer scales in advanced-stage cancer patients. *Journal of Clinical Epidemiology* 2011; **64**(5): 507-16.

6. Cella D, Yount S, Sorensen M, Chartash E, Sengupta N, Grober J. Validation of the Functional Assessment of Chronic Illness Therapy Fatigue Scale relative to other instrumentation in patients with rheumatoid arthritis. *The Journal of rheumatology* 2005; **32**(5): 811-9.

7. Jaeschke R, Singer J, Guyatt GH. Measurement of health status: Ascertaining the minimal clinically important difference. *Controlled Clinical Trials* 1989; **10**(4): 407-15.

8. Zimmerman M, Martinez JH, Young D, Chelminski I, Dalrymple K. Severity classification on the Hamilton depression rating scale. *Journal of Affective Disorders* 2013; **150**(2): 384-8.

9. González-Pinto A, Mosquera F, Reed C, et al. Validity and Reliability of the Hamilton Depression Rating Scale (5 Items) for Manic and Mixed Bipolar Disorders. *The Journal of Nervous and Mental Disease* 2009; **197**(9): 682-6.

10. Buchman AS, Wilson RS, Bennett DA. Total Daily Activity is Associated With Cognition in Older Persons. *The American Journal of Geriatric Psychiatry* 2008; **16**(8): 697-701.

11. Buchman AS, Dawe RJ, Yu L, et al. Brain pathology is related to total daily physical activity in older adults. *Neurology* 2018; **90**(21): e1911-e9.

**References**
